# Catalogue and Price Current

**Published:** 1887-05

**Authors:** 


### BOOK NOTICES.

Catalogue and Price Current of Homœopathic Goods of every description, for sale by Otis Clapp & Son, Boston.

This is a book of two hundred and fifty pages. It contains within its pages a list of absolutely everything a homœopathic physician needs in his practice, even if he be a surgeon. It is copiously illustrated with excellent woodcuts of all kinds of surgical instruments, gynæcological chairs, splints, galvanic and Faradic batteries, medicine cases, especially buggy cases, and vials. There are price lists of remedies, vials, corks, and other things. Not content with including so much, the Messrs. Clapp have added a list of all homœopathic physicians practicing in New England and have not forgotten that indispensable requirement of a good book, a copious index. In issuing this catalogue the Messrs. Clapp announce their removal from No. 3 Beacon Street, to more commodious quarters, at No. 10 Park Square, Boston.

